# Supplementary material for: Synergistic Effect of SiO2 and Fe3O4 Nanoparticles in Autophagy Modulation
Source: Nanomaterials (Basel). 2024 Jun 15;14(12):1033. doi: 10.3390/nano14121033 (PMC11207018; doi:10.3390/nano14121033)
Supplement: Supplementary file 1 [file nanomaterials-14-01033-s001.zip › nanomaterials-3039119-supplementary.pdf]

nanomaterials

Supporting Materials

Synergistic Effect of SiO_2 and Fe_3O_4 nanoparticles in autophagy modulation

Sitansu Sekhar Nanda [1†], Danyeong Kim [2†], Hyewon Yang [2], Seong Soo A. An [2*] and Dong Kee Yi [1*]

1Department of Chemistry, Myongji University, Yongin 17058, Republic of Korea
2Department of Bionanotechnology, Gachon Medical Research Institute, Gachon University, Seongnam, Republic of Korea
† These authors contributed equally.
* Correspondence: vitalis@mju.ac.kr; seongaan@gachon.ac.kr

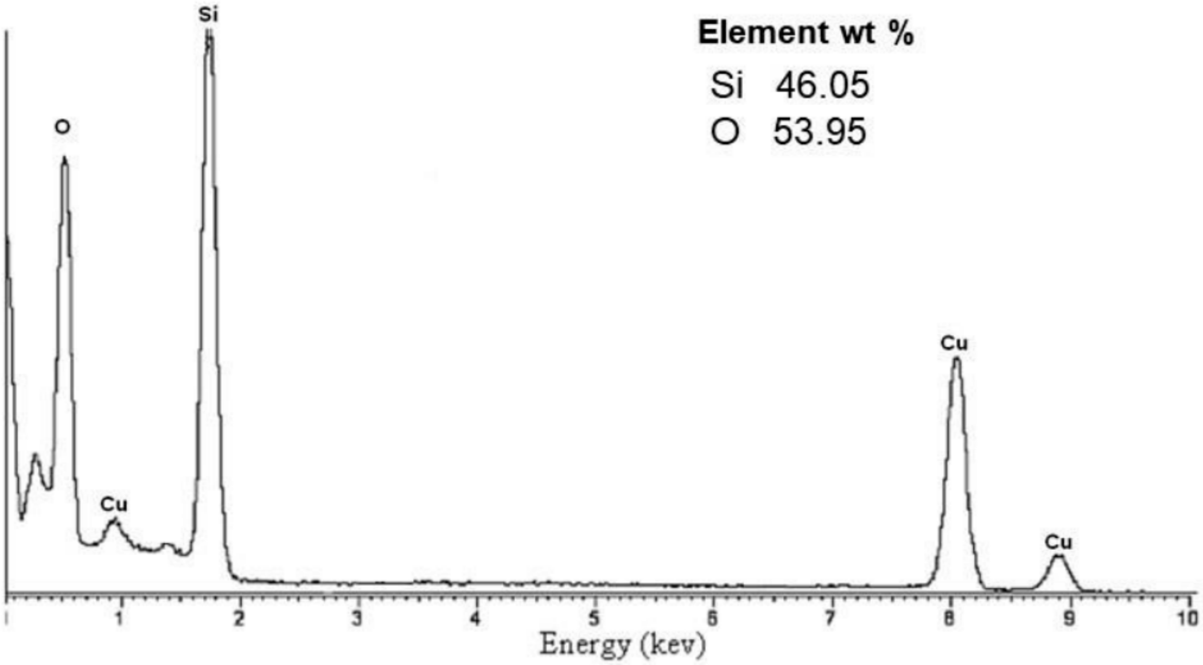

Figure S1. EDAX analysis of SiO_2 nanoparticles.

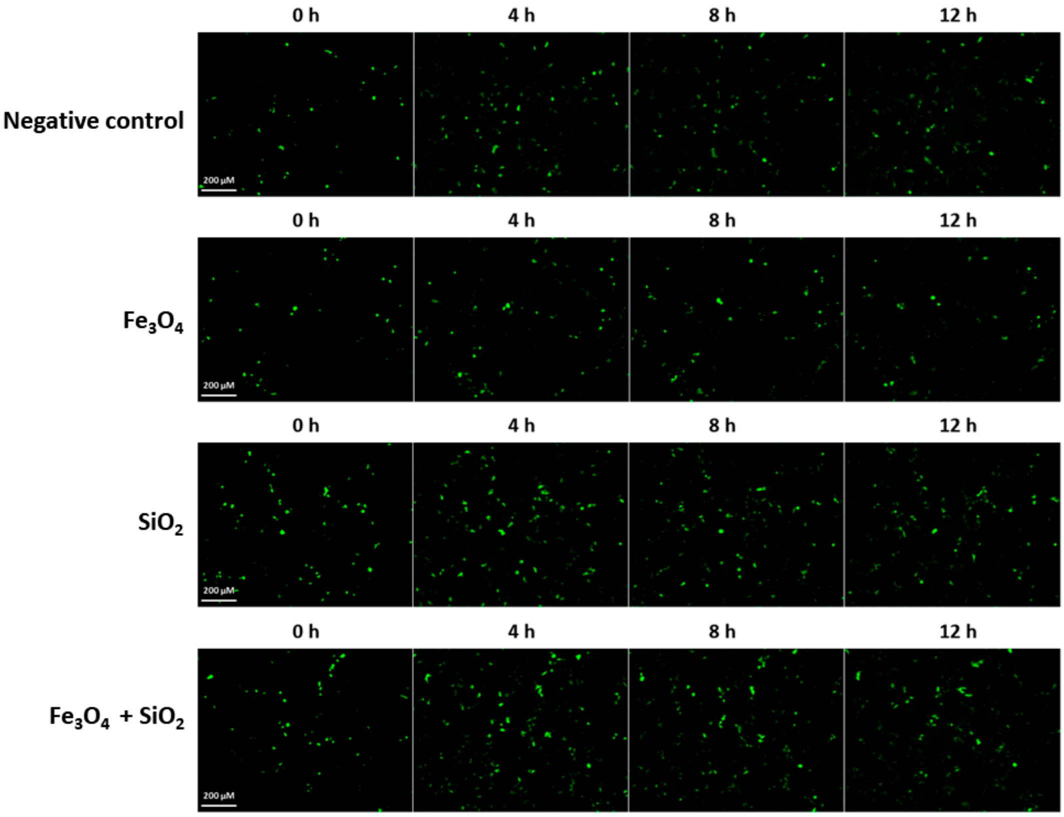

Figure S2. Live change of LC3 expression after treating nanoparticles. Scale bar: 200μm.
